# Acceleration of Small Intestine Development and Remodeling of the Microbiome Following Hyaluronan 35 kDa Treatment in Neonatal Mice

**DOI:** 10.3390/nu13062030

**Published:** 2021-06-12

**Authors:** Hala Chaaban, Kathryn Burge, Jeffrey Eckert, MaJoi Trammell, David Dyer, Ravi S. Keshari, Robert Silasi, Girija Regmi, Cristina Lupu, Misty Good, Steven J. McElroy, Florea Lupu

**Affiliations:** 1 Department of Pediatrics, Division of Neonatology, University of Oklahoma Health Sciences Center, Oklahoma City, OK 73104, USA; kathryn-burge@ouhsc.edu (K.B.); jeffrey-eckert@ouhsc.edu (J.E.); 2 Department of Microbiology and Immunology, University of Oklahoma Health Sciences Center, Oklahoma City, OK 73104, USA; majoi-trammell@ouhsc.edu (M.T.); david-dyer@ouhsc.edu (D.D.); 3 Cardiovascular Biology Research Program, Oklahoma Medical Research Foundation, Oklahoma City, OK 73104, USA; ravi-keshari@omrf.org (R.S.K.); robert-silasiMansat@omrf.org (R.S.); girija-regmi@omrf.org (G.R.); cristina-lupu@omrf.org (C.L.); Florea-Lupu@omrf.org (F.L.); 4 Department of Pediatrics, Washington University School of Medicine, St. Louis, MO 63110, USA; mistygood@wustl.edu; 5 Department of Microbiology and Immunology, Stead Family Department of Pediatrics, University of Iowa, Iowa City, IA 52242, USA; steven-mcelroy@uiowa.edu

**Keywords:** necrotizing enterocolitis, intestinal barrier, human milk bioactive factors, hyaluronan, preterm infants, prebiotics

## Abstract

The beneficial effects of human milk suppressing the development of intestinal pathologies such as necrotizing enterocolitis in preterm infants are widely known. Human milk (HM) is rich in a multitude of bioactive factors that play major roles in promoting postnatal maturation, differentiation, and the development of the microbiome. Previous studies showed that HM is rich in hyaluronan (HA) especially in colostrum and early milk. This study aims to determine the role of HA 35 KDa, a HM HA mimic, on intestinal proliferation, differentiation, and the development of the intestinal microbiome. We show that oral HA 35 KDa supplementation for 7 days in mouse pups leads to increased villus length and crypt depth, and increased goblet and Paneth cells, compared to controls. We also show that HA 35 KDa leads to an increased predominance of Clostridiales Ruminococcaceae, Lactobacillales Lactobacillaceae, and Clostridiales Lachnospiraceae. In seeking the mechanisms involved in the changes, bulk RNA seq was performed on samples from the terminal ileum and identified upregulation in several genes essential for cellular growth, proliferation, and survival. Taken together, this study shows that HA 35 KDa supplemented to mouse pups promotes intestinal epithelial cell proliferation, as well as the development of Paneth cells and goblet cell subsets. HA 35 KDa also impacted the intestinal microbiota; the implications of these responses need to be determined.

## 1. Introduction

Necrotizing enterocolitis (NEC) is a devastating neonatal gastrointestinal disease primarily affecting preterm infants [1,2,3]. While the etiology is unclear, evidence suggests that NEC is a multifactorial process, with intestinal immaturity, a dysregulated immune system, and an altered intestinal microbiome playing major roles in the pathogenesis of the disease. The intestine of preterm infants also displays impaired barrier function with increased intestinal permeability, prominent intestinal epithelial Toll-like receptor 4 (TLR4) expression, and an increased abundance of pathogenic bacteria [4,5,6,7]. Therefore, identifying strategic interventions that promote postnatal maturation of the intestinal epithelial barrier and hasten the development of a favorable microbiome is critical for protecting this population.

The protection in preterm infants against NEC afforded by human milk (HM) has long been established [8,9,10,11,12,13]. Human milk (containing bioactive compounds such as lactoferrin, immunoglobulin, and HM oligosaccharides) decreases intestinal permeability and enhances the underdeveloped physical barrier [13,14,15,16,17,18]. HM is also rich in hyaluronan (HA), a uniquely nonsulfated glycosaminoglycan (GAG) with repeating units of D-glucuronic acid and N-acetyl-D-glucosamine [19]. HA is produced by membrane-bound HA synthases 1–3 (HAS1-3) and can have either pro- or anti-inflammatory properties depending upon the size, mode of administration, and physiological milieu in which the GAG is deposited [20]. Notably, oral administration of either HA purified from HM or biosynthetic HA of an average molecular weight of 35kDa (HA 35 KDa), a HM mimic, protects against bacterial-induced colitis by increasing the expression of antimicrobial β-defensins and tight junction (TJ) protein expression in the colonic epithelium [21,22,23,24]. Our previous data in a murine model of NEC demonstrated the ability of oral HA 35 KDa to reduce mortality, lessen the severity of the intestinal injury, and decrease systemic inflammation, partly through preservation of the intestinal barrier function [25].

Importantly, peak concentrations of HM HA are produced during the first weeks following birth, suggesting a role for HA in intestinal development and protection against enteric pathogens during this critical period [21]. Similar to other GAGs, HA reaches the human colon undigested, allowing for substantial interaction with the developing infant microbiome [26,27]. Given the demonstrated protective effects against both NEC and colitis, we hypothesized that HA 35 KDa may promote postnatal intestinal growth and differentiation, as well as alter the intestinal microbiome. Our novel data show that HA 35 KDa supplementation increases intestinal villus length, crypt depth, and number of secretory intestinal epithelial cells. Additionally, our data indicate that HA 35 KDa supplementation increases the predominance of the beneficial Clostridiales Ruminococcaceae, Lactobacillales Lactobacillaceae, and Clostridiales Lachnospiraceae in the gut microbiome. Furthermore, ileal transcriptome analysis identified the enrichment of several genes essential for growth, proliferation, and survival in pups supplemented with the HA 35 KDa group. These differentially-expressed genes include human epidermal growth factor receptor 2 (HER-2), genes associated with the ERK/MAPK (extracellular signal-regulated kinase/mitogen-activated protein kinase), mTOR (mechanistic target of rapamycin), IGF-1 (insulin-like growth factor 1), and PI3K/AKT (phosphatidylinositol 3-kinase/protein kinase B) pathways. Altogether, our data demonstrate that HA, a critical component of early HM, speeds postnatal maturation of the intestine and may serve as a developmental cue to promote a healthy microbiome.

## *2.* Materials and Methods

### 2.1. Mouse Experiments

**Figure 1:**
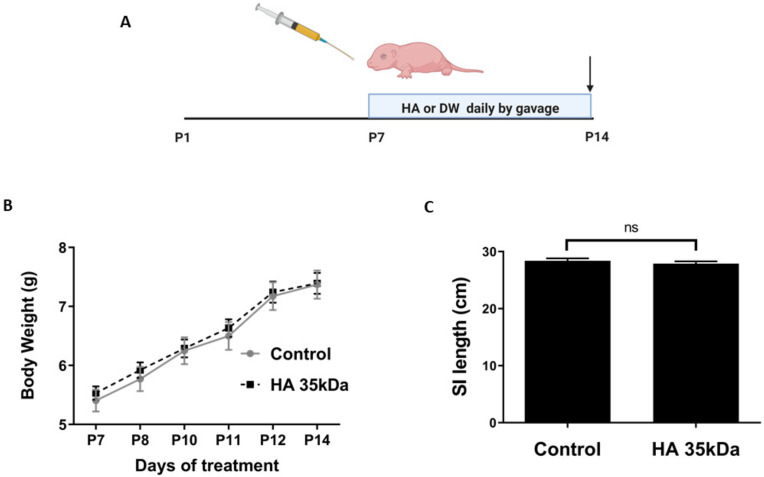
Oral HA 35 KDa supplementation for 7 days does not affect pup body weight or small intestinal lengthening. (**A**) Mouse pups were gavaged either HA 35 KDa (30 mg/kg) or an equal volume of sterile water daily from P7–P14, with arrow depicting the end of treatment. (**B**) Pups from both groups (*n* > 10 mice per group) were weighed daily during supplementation. HA 35 KDa did not affect the weight gain compared to controls (*p* ≥ 0.574). (**C**) SI length at the time of euthanasia (P14) did not differ between the groups (*p* = 0.394). Data presented as mean ± SEM. HA: hyaluronic acid; PBS: phosphate-buffered saline; SI: small intestine; SEM: standard error of the mean.

All animal studies were approved by the Institutional Animal Care and Use Committee at the University of Oklahoma Health Sciences Center (IACUC number:19-062-EFCHI). Timed pregnant CD-1 dams (Charles River Laboratories, Wilmington, MA, USA) were housed individually under a 12 h dark/light cycle in sterilized filter-top cages and provided ad libitum food and water. Pups born vaginally of both sexes were weighed daily and randomized into groups receiving HA 35 KDa (Lifecore Biomedical, Chaska, MN, USA) or the control at day P7 of life. HA 35 KDa is pure biosynthetic, medical grade, commercially manufactured to meet the European Pharmacopoeia and Japanese Pharmacopoeia monographs for sodium hyaluronate. Body weights were recorded daily, and pups received either 30 mg/kg HA 35 KDa or an equal volume of sterile water via oral gavage daily for 1 w (P7–P14) (Figure 1A). The dose and duration of treatment were based on previous experiments [25]. At the end of treatment, pups from both groups were anesthetized with isoflorane and euthanized via bilateral thoracotomy and cardiac puncture. After measuring small intestinal length, sections from terminal ileal tissue were harvested in RNAlater (Invitrogen, Carlsbad, CA, USA) for gene expression analysis, or fixed in either 10% buffered formalin or Carnoy’s solution (60% ethanol, 30% chloroform, 10% glacial acetic acid) as appropriate for histology and immunohistochemical staining.

### 2.2. Histology

Serial histological sections of 5 µm thickness were cut, deparaffinized, rehydrated, and stained with hematoxylin and eosin (H&E) for morphological analysis. Utilizing ImageJ (National Institutes of Health, Bethesda, MD, USA), no fewer than 50 villus heights and crypt depths were measured per animal, with at least 3 animals analyzed per group. Crypts were counted strictly from well-oriented sections with intact bilateral crypt-villus junctions. Villi were counted only from sections showing the central lymphatic channel extended from villus base to tip. For determination of Paneth cell and goblet cell numbers, sections were stained with Periodic Acid Schiff (PAS)/Alcian blue (AB) (MilliporeSigma, St. Louis, MO, USA), as described [28].

### 2.3. Immunohistochemical Staining

Paraffin-embedded sections were deparaffinized, rehydrated, then steamed for 30 min in citrate buffer for antigen retrieval, after blocking 5% goat serum in Tris-buffered saline with 0.05% Tween 20. Sections were incubated with primary antibodies against either Ki-67 (1:100, ThermoFisher Scientific, Waltham, MA, USA) or Phospho-S6 Ribosomal Protein (1:400, Cell Signaling Technology). Horse-radish peroxidase-conjugated secondary antibodies (rabbit anti-goat IgG 1:200, Abcam, Cambridge, MA, USA, and goat anti-rabbit IgG, 1:100: Abcam) followed by the SignalStain DAB Substrate Kit (Cell Signaling Technology) were used for detection. Nuclear counterstaining was performed using hematoxylin. For apoptosis detection, ileal sections were stained with terminal deoxynucleotidyl transferase dUTP nick end labeling (TUNEL) using the In Situ Cell Death Detection Kit, TMR red (Roche Diagnostics, Indianapolis, IN, USA), per manufacturer’s instructions. Sections were mounted with VECTASHIELD Antifade Mounting Medium (Vector Laboratories, Burlingame, CA, USA) containing DAPI (4′,6-diamidino-2-phenylindole) as a nuclear stain. Slides were visualized with the C1 laser scanning Confocal Microscope System (Nikon, Melville, NY, USA), and analyzed using ImageJ.

### 2.4. 16S Ribosomal RNA (rRNA) Sequencing and Microbiome Analysis

Identification and analysis of pup bacterial composition of the cecum was performed by the Microbiome and Transcriptomics Core at OUHSC. The cecal contents of 41 pups were collected in bead tubes from the Stool DNA Isolation Kit (Norgen Biotek Corp., Thorold, ON, Canada) following euthanasia, and immediately snap-frozen in liquid nitrogen. Samples were subsequently transferred for storage at −80 °C until further analysis. Bacterial DNA extraction was performed according to manufacturer’s instructions. DNA quantity and quality were characterized by the NanoDrop Lite Spectrophotometer (ThermoFisher Scientific). PCR amplification of the hypervariable V3–V4 segment of the 16S rRNA gene was achieved using an Illumina kit. Libraries were sequenced on the Illumina MiSeq platform using the MiSeq Reagent Kit v3 for 600 cycles to collect 300 bp paired-end reads. The generated data (up to 18 Gb) were analyzed using QIIME2 [29]. Sequencing reads were classified against the Greengenes 16S rRNA database [30] using amplicon sequence variants (ASVs). PERMANOVA was employed to determine the β-diversity distances within and between groups, and Kruskal–Wallis was used to determine the statistical significance of α-diversity. Phylogenetic Investigation of Communities by Reconstruction of Unobserved States (PICRUSt) analysis was performed from EC numbers grouped into MetaCyc reactions to identify metabolic pathways potentially affected by groups of bacteria [31]. Differential abundance analysis utilized the nonparametric permutation difference test for α-diversity and PICRUSt values or the negative binomial test for taxonomic count data. *p*-values were then adjusted using FDR.

### 2.5. RNA Extraction and qPCR Analysis

Ileal sections collected in RNAlater and stored at −80 °C were homogenized with a 7 cm Polypropylene Pellet Pestle (W.W. Grainger, Lake Forest, IL, USA), passed through a QIAshredder homogenizer (Qiagen, Germantown, MD, USA), and subjected to total RNA isolation using a RNeasy Plus Mini Kit (Qiagen, USA). Sample quantity and quality were assessed using a NanoDrop Lite Spectrophotometer (ThermoFisher Scientific), and 1 µg RNA was reverse transcribed to cDNA using a High Capacity cDNA Reverse Transcription Kit (Applied Biosystems, Foster City, CA, USA). qPCRwas performed on a StepOne Real-Time PCR System (Applied Biosystems) using TaqMan Fast Universal PCR Master Mix (2X) (Applied Biosystems) and TaqMan Gene Expression Assays from Applied Biosystems for *muc2* (Mm00458307_g1), *lyz11* (Mm00657323_m1), *Eef4e* (Mm00725633_s1), *Rheb* (Mm00474045_m1), *Mtor* (Mm00444968_m1), and *Rps27a* (Mm01180369_g1). Samples were run in triplicate from 10 pups in both groups. Data were analyzed using the 2^−ΔΔCt^ method, with fold changes normalized to the housekeeping *Gadph* (Mm99999915_g1) gene.

### 2.6. Host RNA-Seq Profiling and Enrichment Analysis

Stranded RNA-Seq libraries were constructed for 4 animals per group using the CORALL Total RNA-Seq Library Prep Kit v2 (Lexogen, Greenland, NH, USA) following established protocols. Library construction was completed with 200–500 ng of RNA. Each library was indexed during library construction to multiplex for sequencing. Samples were normalized, and 8 libraries were pooled and run on the Illumina NovaSeq 6000 Platform (San Diego, CA, USA) at the Oklahoma Medical Research Foundation Clinical Genomics Center. Derived sequences were analyzed by applying a custom computational pipeline consisting of the open-source gSNAP, Cufflinks, and R for sequence alignment and ascertainment of differential gene expression [32]. Reads generated were mapped to the mouse genome (mm10) by gSNAP [33], expression (fragments Per Kilobase of transcript per Million mapped reads, FPKM) derived by Cufflinks [34], and differential expression analyzed with ANOVA in R. The log2 fold change of genes significant at a false discovery rate (FDR) <0.05 were submitted to pathway analysis using Ingenuity Pathway Analysis (IPA) software (Qiagen) to identify pathways of interest from the dataset associated with the Ingenuity Knowledge Base repository (Ingenuity Systems, Inc., Redwood City, CA, USA). IPA Functional Analysis was used to identify biological functions and/or diseases significantly associated with the dataset. Fisher’s exact test was used to calculate a *p*-value determining the probability of the association between genes in the dataset and the pathway of interest compared to chance alone. To assess the activation state of a pathway and upstream regulators, the consistency of the match between the observed and predicted pattern was calculated (Z-score). A positive Z-score signifies a regulator in the active state, while a negative Z-score indicates repressed activity. Canonical pathway analysis was used to identify networks from the IPA library most significantly modulated across both groups. The significance of the association between each dataset and the canonical pathway was measured as a ratio of the number of molecules from the dataset mapped to the pathway divided by the total number of molecules mapped to the canonical pathway.

### 2.7. Statistical Analysis

Statistical analysis was performed using GraphPad Prism version 6 (GraphPad Software, La Jolla, CA, USA). Data are presented as mean ± standard error of the mean (SEM). Unless otherwise noted, data were analyzed by unpaired *t*-tests or the Mann–Whitney test, as appropriate. A *p*-value less than 0.05 was considered significant.

## 3. Results

### 3.1. Effect of Oral HA 35 KDa on Pup Weight Gain and Intestinal Lengthening

The development of the mouse pup intestine from P7–P14 corresponds with that of the human intestine at approximately 22–23 weeks gestation, the most susceptible period for NEC development in preterm human infants [35]. Therefore, we first sought to evaluate whether HA 35 KDa supplementation during P7–P14 affected pup weight gain and small intestinal lengthening. Pups were randomized to receive either 30 mg/kg HA 35 KDa or an equal volume of sterile PBS by gavage once daily (Figure 1A). As shown in Figure 1B, HA 35 KDa treatment did not affect pup weight gain from P7–P14 compared to controls, with HA 35 KDa pups gaining an average of 0.372 g/day and controls, 0.395 g/day (*p* ≥ 0.574). Upon euthanasia, the overall gross length of the small intestine (Figure 1C) did not differ between the groups (HA 35 KDa: 27.88 ± 1.4cm; Control: 28.39 ± 1.25cm; *p* = 0.394).

### 3.2. Effect of Oral HA 35 KDa on Villi Length, Crypt Depth, Intestinal Epithelial Proliferation, and Apoptosis, and Differentiation of Secretory Epithelium

Previous studies have shown that intraperitoneal administration of high molecular weight HA (750 kDa) promotes epithelial proliferation and intestinal development [36,37,38]. Considering the demonstrated benefits of HA 35 KDa in ameliorating NEC [25], we hypothesized that oral HA 35 KDa would affect the small intestinal development in mouse pups.

**Figure 2:**
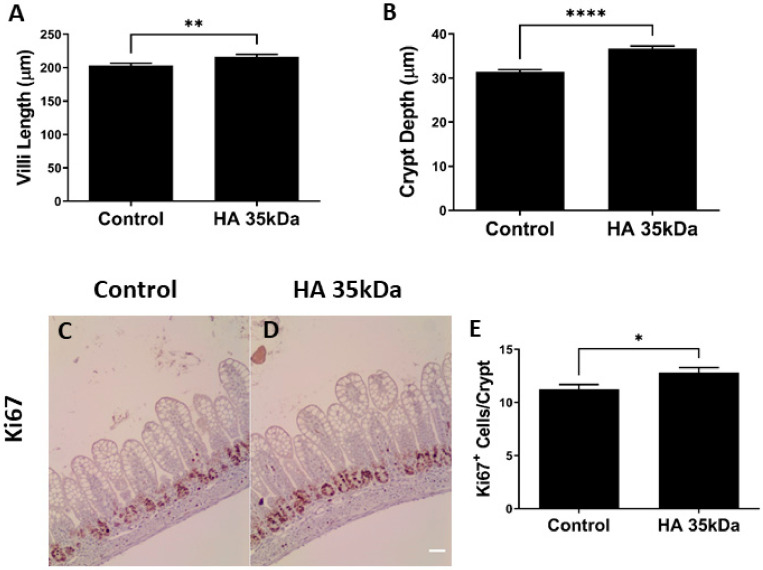
Oral HA 35 KDa supplementation from P7–P14 increases villi length (**A**) and crypt depth (**B**) in mouse pups (*n* = 206 villi/crypts; *p* = 0.0085 (illi length) *p* < 0.0001 (crypt depth). A comparison of Ki67 staining (brown) of the ileum in controls (**C**) and HA 35 KDa (**D**) pups indicates increased intestinal epithelial proliferation with oral HA 35 KDa. Quantification (*n* = 131 crypts; *p* = 0.0175) of Ki67 staining in (**E**). Data presented as mean ± SEM. HA: hyaluronic acid; * *p* < 0.05; ** *p* < 0.01; **** *p* < 0.0001.

Pups treated with HA 35 KDa exhibited increased ileal villus length (HA 35 KDa: 216.3 µm; Control: 203.4 µm; *p* = 0.0085) and crypt depth (HA 35 KDa: 36.72 µm; Control: 31.38 µm; *p* < 0.0001) compared to controls (Figure 2A,B). Consistent with these results, pups in the HA 35 KDa group had enhanced Ki67 staining (HA 35 KDa: 12.8 cells/crypt; Control: 11.24 cells/crypt; *p* = 0.0175), indicative of increased cell cycle progression to mitosis and consistent with intestinal epithelial proliferation (Figure 2C–E). To determine the contribution of apoptosis in the observed changes, TUNEL staining was performed on ileal sections from both groups (data not shown). No differences in the number of apoptotic cells were noted between the groups, suggesting the more advanced development of the intestine in HA 35 KDa pups could be attributed to an increase in proliferation rather than a decrease in apoptosis.

**Figure 3:**
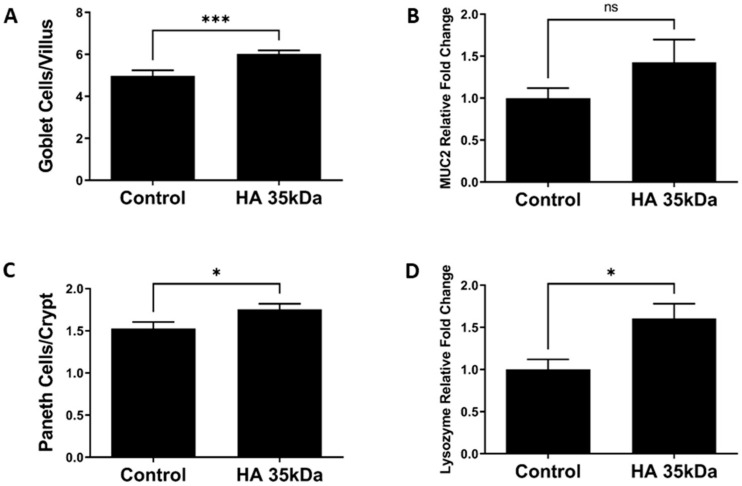
Effects of oral HA 35 KDa supplementation on goblet cell number per villus (**A**; *n* = 168 villi; *p* = 0.0009), MUC2 mRNA expression (**B**; *n* = 20; *p* = 0.5027), Paneth cell number per crypt (**C**; *n* = 162 crypts; *p* = 0.0247), and lysozyme mRNA expression (**D**; *n* = 22; *p* = 0.0127) in ileal tissue from both groups. Data presented as mean ± SEM. HA: hyaluronic acid; MUC2: mucin 2; SEM: standard error of the mean. * *p* < 0.05, *** *p* < 0.001, ns = not significant.

Next, we determined the effect of oral HA 35 KDa on differentiation of the intestinal Paneth and goblet cells. Compared to control pups, the number of goblet cells per villus represented as PAS/AB positive cells was significantly higher in HA 35 KDa-treated animals (Figure 3A; HA 35 KDa: 6.023 cells/villus; Control: 4.971 cells/villus; *p* = 0.0009). Paneth cell number per crypt also increased following HA 35 KDa treatment compared to controls (Figure 3C; HA 35 KDa: 1.756 cells/crypt; Control: 1.528 cells/crypt; *p* = 0.0247). Further evidence of the increase in differentiated secretory cell types was provided by gene expression (Figure 3B,D) for goblet cell mucin 2 (HA 35 KDa: 1.427; Control: 1; *p* = 0.5027) and Paneth cell lysozyme (HA 35 KDa: 1.606; Control: 1; *p* = 0.0127). Altogether, these data suggest that oral HA 35 KDa supplementation increases intestinal epithelial proliferation and differentiation of secretory cell types.

### 3.3. Effect of Oral HA 35 KDa on Intestinal Microbiome Taxonomy and Functional Prediction

**Figure 4:**
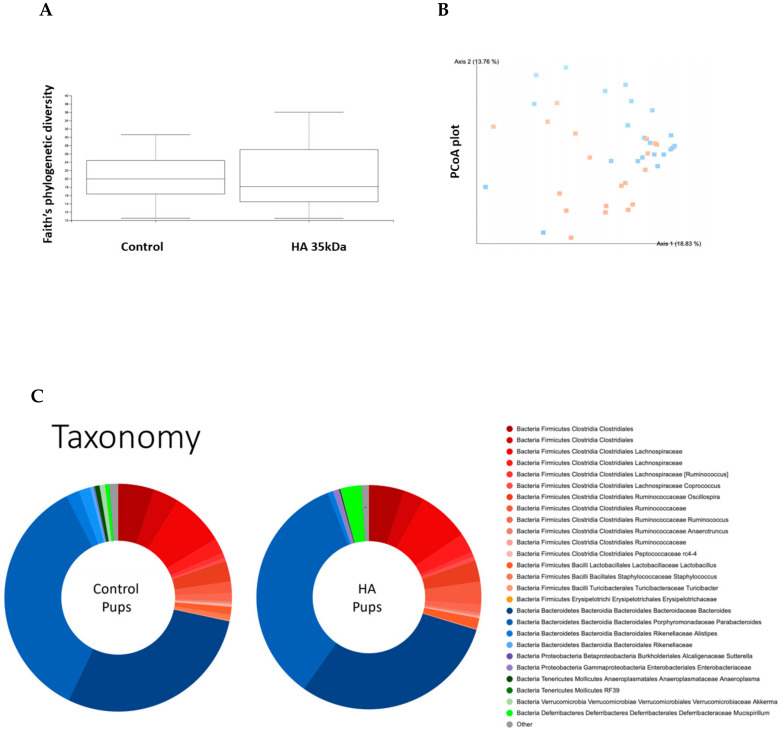
Analysis of cecal microbiota composition between groups. (**A**) Faith’s phylogenetic diversity metric displaying species richness of mouse pups differentiated by treatment (*n* = 41; Kruskal–Wallis *p* = 0.69). (**B**) PCoA plot displaying Bray Curtis beta diversity matrix (Control = orange; HA 35 KDa = blue). Percent confidence values for each distance matrix are displayed on the axes in two dimensions. PERMANOVA results: *F* = 3.10; *p* = 0.002. (**C**) Taxonomy plots generated using a Naïve–Bayes classifier trained on the most recent Greengenes 16S rRNA database. Amplicon sequence variance (ASV) reads were taxonomically classified and filtered for genera >0.5% of total microbiome composition for any one sample. Genera falling below the 0.5% mark were placed in “other” (shown in grey). HA: hyaluronic acid; PCoA: principal components analysis.

The effects of HA 35 KDa treatment on the developing microbiome in mouse pups are unknown. Thus, we evaluated the effect of HA 35 KDa on the cecal bacterial composition at day P14. A total of 24,767,410 16S rRNA amplified sequences were obtained from 41 samples. No statistically significant differences were noted in the alpha diversity of microbiota between groups (Figure 4A; Kruskal–Wallis *p* = 0.69). Nevertheless, principal coordinates analysis (PCoA) showed significant clustering by the group based on the Bray Curtis beta diversity matrix (Figure 4B; PERMANOVA: *F* = 18.52, *p* = 0.001), suggesting significant dissimilarity between bacterial communities of control and HA 35 KDa-treated pups. As expected, the taxonomic composition of the gut microbiomes of both control and HA 35 KDa-treated pups was dominated primarily by Firmicutes and Bacteroides phyla (Figure 4C).

**Figure 5:**
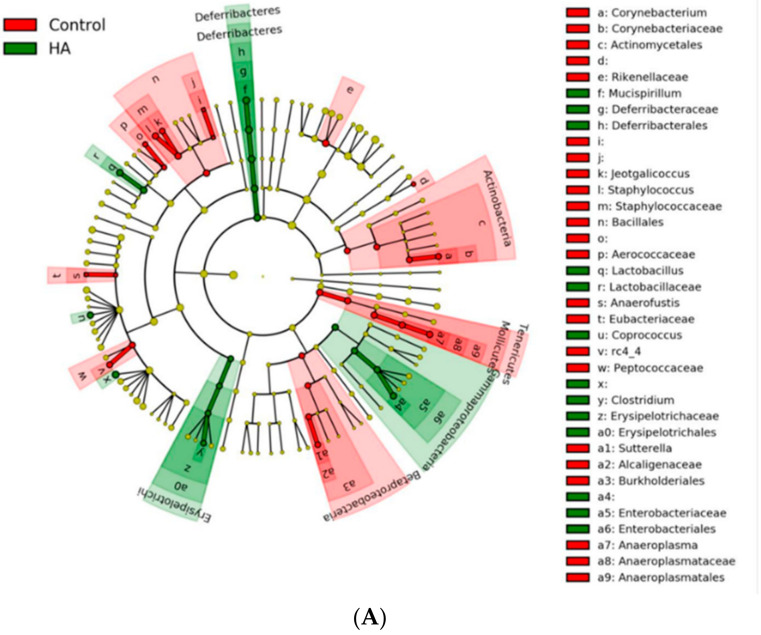

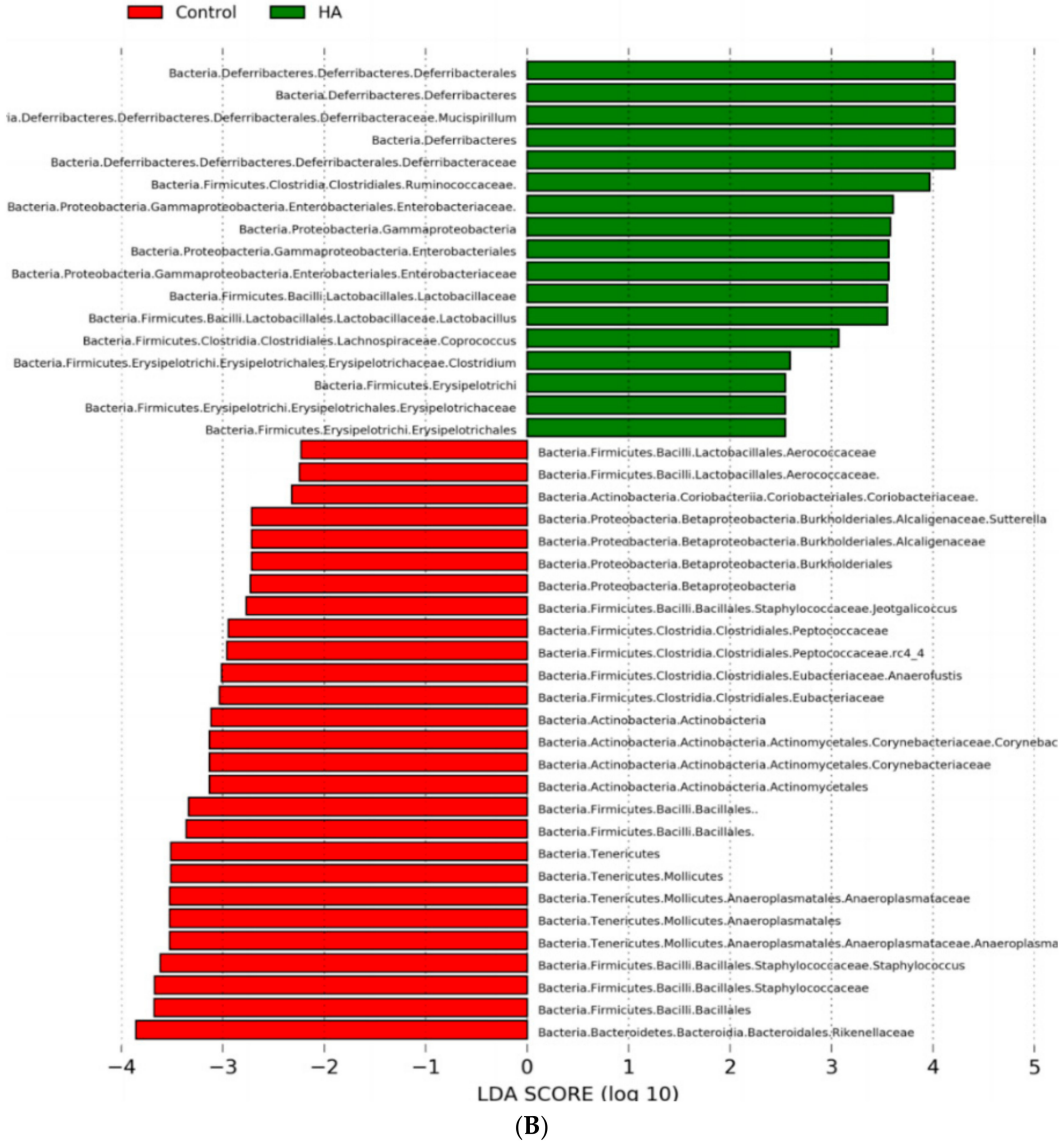
LEfSe analysis between control and HA 35 KDa groups. (**A**) Cladogram utilizing LEfSe indicates phylogenetic distribution of fecal microbes associated with control (green) and HA 35 KDa-treated (red) pups. (**B**) LDA scores of significantly different bacteria between control (red) and HA 35 KDa-treated (green) pups. HA: hyaluronic acid; LEfSe: linear discriminant analysis effect size; LDA: linear discriminant analysis.

Comparisons using linear discriminant analysis (LDA) effect size (LEfSe) revealed several differences between the two groups’ microbial populations at the phylum, class, family, genus, and species levels. HA 35 KDa-treated pups had an increased abundance at the family level of *Deferribacterales Deferribacteraceae*, *Clostridiales Ruminococcaceae*, *Enterobactereriales Enterobactereriaceae*, *Lactobacillales Lactobacillaceae*, and *Clostridiales* *Lachnospiraceae*. In contrast, the cecal microbiome of control pups contained a preponderance of *Bacillales*, *Staphylococcaceae*, *Bacteroidales Rikenellaceae*, *Actinomycetes Corynebacteriaceae*, among others (Figure 5B; LDA score (log 10) > 2).

**Figure 6:**
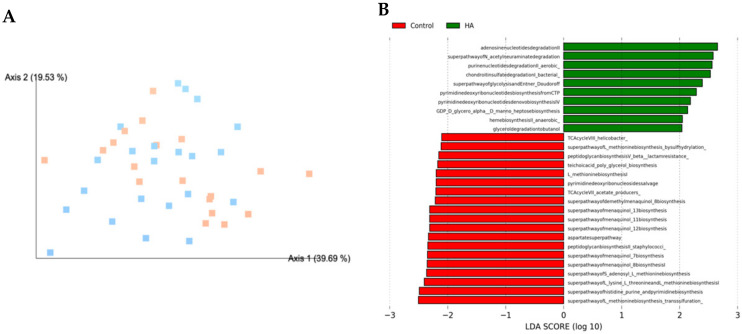
PICRUSt2 functional prediction. (**A**) PCoA plot displaying Bray Curtis beta diversity matrix calculations (Control = orange; HA 35 KDa = blue). Percent confidence values for each distance matrix are displayed on the axes in two dimensions. PERMANOVA results: *F* = 1.80; *p* = 0.1. (**B**) Predicted metabolic pathways statistically attributed to the experimental variable. PICRUSt was used to predict the functional potential of microbiomes using 16S rRNA gene sequence data. Red bars represent controls; green bars represent HA 35 KDa-treated pups. HA: hyaluronic acid; PCoA: principal components analysis; LDA = linear discriminant analysis; PICRUSt = Phylogenetic Investigation of Communities by Reconstruction of Unobserved States.

Based on significant bacterial phylogenetic distribution differences, we then used PICRUSt analysis to predict the MetaCyc metabolic pathways potentially differing between control and HA 35 KDa-treated pups. Clustering of microbial communities based on gene content revealed a similar pattern in both groups (Figure 6A). However, LEfSe analysis of PICRUSt output suggested several differences in the predicted biological pathways between HA 35 KDa-treated and control pups (LDA score ≥ 2.0). Specifically, 29 MetaCyc pathways significantly diverged between the microbiomes of HA 35 KDa-treated and control pups. HA 35 KDa-treated pups appear to have differentially enriched metabolic pathways involved in the degradation of adenosine and purine nucleotides, sialic acid, chondroitin sulfate, and butanol. In addition, pathways for Entner–Doudoroff glycolysis, and the synthesis of pyrimidine deoxyribonucleotide, heme and heptose sugars are predicted to be upregulated in HA 35 KDa pups. In contrast, the cecal microbiome of control pups was enriched for biosynthetic pathways for amino acids, such as methionine and histidine, nitric oxide reductase and cofactor synthesis, and biosynthesis of bacterial cell wall components including peptidoglycan II and V (β-lactam resistance), Figure 6B.

### 3.4. Effect of HA 35 KDa on Ileal Transcriptional Profiles Using RNA-Seq Analysis

To define the effects of HA 35 KDa on the ileal transcriptome, bulk RNA-Seq analysis was performed on tissues from the controls and HA-treated groups. Using a significance cutoff of *p* ≤ 0.05, a total of 247 differentially expressed genes (DEGs; 200 upregulated and 47 downregulated) were identified in HA 35 KDa-treated pups compared to controls (Supplementary Materials Table S1).

**Figure 7:**
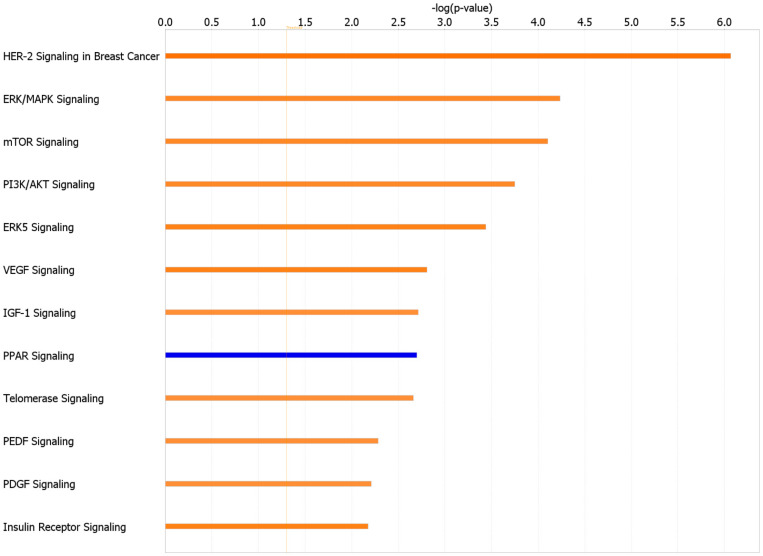
Top canonical pathways in terminal ileum affected by HA 35 KDa supplementation, ranked by Z-score with a cutoff ≥ 2 (orange, activated) and ≤−2 (blue, inhibited).

**Figure 8:**
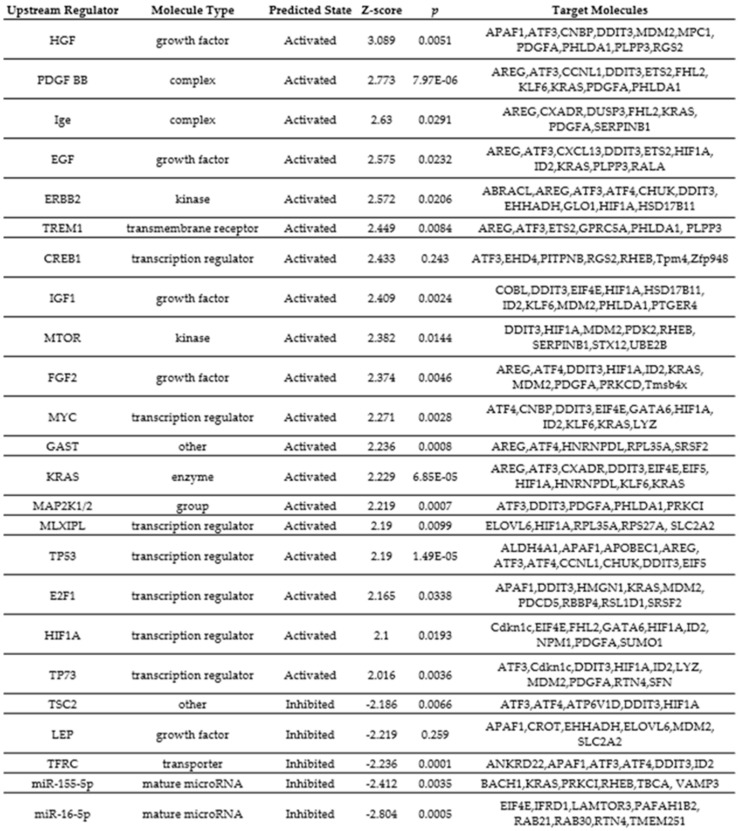
Top biological processes in HA 35 KDa-treated pups compared to controls. The predicted GO categories were ranked by enrichment score (−log (*p*-value)) HA: hyaluronic acid; GO: gene ontogeny.

We next used IPA to map DEGs to disease-related gene ontology (GO) pathways and predict upstream transcriptional regulators mediated by the observed transcriptional networks. The top canonical pathways, with Z-scores greater than 2 or less than −2, associated with HA 35 KDa compared to control are listed in Figure 7. Notably, pathways related to cellular growth, proliferation, and survival, including HER-2, ERK (5)/MAPK, mTOR, IGF-1, and PI3/AKT pathways, were upregulated in the HA 35 KDa group. Similarly, the top-predicted upstream regulators in the HA 35 KDa group include hepatocyte growth factor (HGF), platelet-derived growth factor-subunit BB (PDGF-BB), epidermal growth factor (EGF), Erb-B2 receptor tyrosine kinase 2 (ERBB2), cyclic AMP-responsive element-binding protein 1 (CREB1), mTOR, hypoxia-inducible factor 1-alpha (HIF-1α), IGF-1, Myc, K-Ras, fibroblast growth factor 2 (FGF2), and others. In contrast, tuberous sclerosis complex 2 (TSC2), a negative regulator of the mTOR pathway, miR155-5p and miR16-5pm, transferrin receptor (TFRC), and leptin (LEP) were predicted to be downregulated in the HA 35 KDa group (Supplementary Materials Figures S1 and S2). To identify biological processes predicted to be associated with these pathways in the small intestine further, we utilized the IPA disease and functions analysis with a Z-score cutoff of 2. The predicted processes in the HA 35 KDa mouse pups are related to cellular development, cellular growth and proliferation, and organismal survival. Conversely, GO-disease-related processes associated with organismal death, mortality, and growth failure were predicted to be downregulated in the HA 35 kDa pups compared to controls (Figure 8; Supplementary Materials Table S2).

### 3.5. Oral HA 35 KDa Activates mTOR Pathway in Mouse Pup Ileum

**Figure 9:**
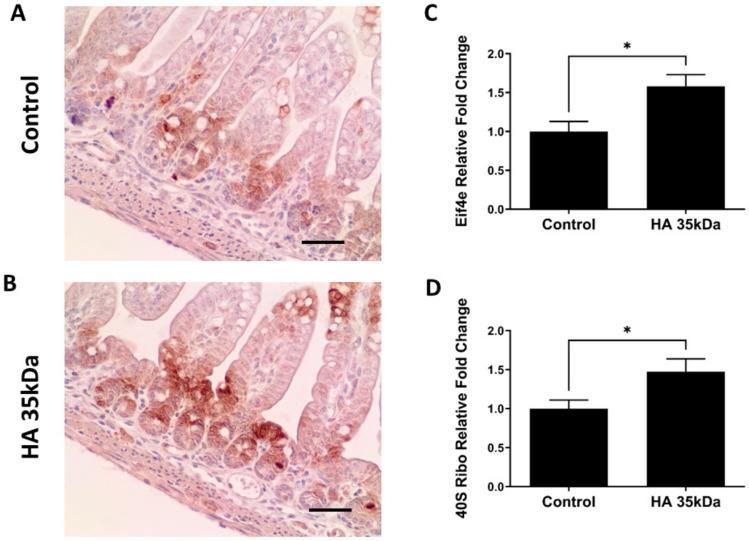
Oral HA 35 KDa activates the mTOR pathway within the intestinal epithelium of P14 pups. Representative immunohistochemical staining for phosphorylation of ribosomal protein S6 in ileal sections from controls (**A**) and HA 35 KDa-treated pups (**B**) Scale bars = 50μm. Confirmatory mRNA expression for *Eif4e* (**C**) and *40S Ribo* (**D**) (*n* = 21). Data presented as mean ± SEM. HA: hyaluronic acid; Eif4e: eukaryotic translation initiation factor 4e; mTOR: mechanistic target of rapamycin; SEM: standard error of the mean. * *p* < 0.05.

Based on the number of IPA-predicted processes involved in cellular proliferation and development of the ileum of pups treated with HA 35 KDa, we interrogated specific proteins and genes involved in the mTOR pathway, one of the most frequently referenced pathways in comparing HA 35 kDA-treated pups to controls (Supplementary Materials Figure S3). mTOR complex 1 (mTORC1), a key component of the mTOR signaling pathway, is a regulator of cell proliferation, maintenance of the intestinal epithelium, and differentiation of secretory cells [39]. Immunostaining intensity of phosphorylation of ribosomal protein S6 (40S Ribo), a common surrogate for mTORC1 activity, was increased in the ileal epithelium of pups treated with HA 35 KDa (Figure 9A,B), particularly in the crypts and transit-amplifying zones. In addition, q-PCR performed for DEGs within the mTOR pathway revealed that mRNA expressions of both Eif4e (eukaryotic translation initiation factor 4e, a downstream component of the mTOR pathway) and 40S Ribo were significantly upregulated in the HA 35 KDa-treated pups (Figure 9C,D; Eif4e: HA 35 KDa: 1.56-fold normalized to control; *p* = 0.01; and 40S Ribo: HA 35 KDa: 1.477 fold normalized to control; *p* = 0.016). Taken together, these data suggest that HA 35 KDa treatment in mouse pups increases mTORC1 activation in the intestinal epithelium.

## 4. Discussion

The intestinal epithelial barrier consists of structural and biochemical defenses that ensure the containment of undesirable luminal contents while preserving the ability to absorb nutrients. Several factors contributing to intestinal barrier integrity are underdeveloped in preterm infants [4,5,40]. Goblet cells secrete mucins, predominantly MUC2, forming a semipermeable layer between the lumen and intestinal epithelium [41]. In addition, the mucous layer contains antimicrobial peptides secreted by enterocytes and Paneth cells [42,43]. Intestinal integrity is reinforced further by tight junction (TJ) proteins, which seal the intercellular space between enterocytes and regulate paracellular permeability [5]. While significant development of the intestinal barrier occurs in utero, postnatal maturation is crucial, especially when infants are born prematurely [5,44]. Preterm Paneth cells and goblet cells are deficient in both number and function [45,46,47,48]. Altered localization and reduced expression of TJ proteins in preterm infants predispose the intestine to abnormal translocation of pathogenic or commensal bacteria [4,49,50]. Moreover, intestines of preterm infants demonstrate delayed commensal colonization, low bacterial diversity, and increased pathogenic bacteria compared to those of term infants [4,5], further predisposing this fragile population to NEC [51,52].

Hyaluronic acid, an abundant extracellular matrix component in vertebrates, is composed of a linear polymer of repeating glucuronic acid and *N*-acetylglucosamine disaccharides [53]. The biological properties of HA can be either pro- or anti-inflammatory depending upon the size, mode of administration, abundance, and downstream receptors, and physiological environment [20]. For example, intraperitoneal administration of HA 750kDa ameliorated intestinal inflammation and injury in a mouse model of dextran sodium sulfate (DSS)-induced colitis [54]. Treatment of mice subjected to whole-body radiation with HA 750kDa also protected against enterocyte apoptosis and was associated with an increased number of intestinal villi compared to controls [55]. Lower molecular weight HA appears to be protective against intestinal infection and inflammation when given orally. Hill et al. demonstrated that oral HA, purified from HM, increased the expression of human β-defensin 2 (hBD-2) and protected against *Salmonella* infection in vivo and in vitro [21,56]. HA 35 KDa appears to mimic the effects of bulk HM HA, increasing expression of mBD-3, the murine equivalent of hBD-2, and modulates epithelial zonula occludens-1 (ZO-1) expression, preventing bacterial translocation in multiple models of colitis [20,57]. Our recent data indicate HA 35 KDa is also protective in a murine model of NEC, reducing incidence, severity, and mortality in the intraperitoneal dithizone/oral *Klebsiella pneumoniae* NEC model [25,46]. In addition, growing evidence supports the ability of endogenous HA to enhance the normal development of the gut [38,57]. Neonatal mouse pups treated from P7–14 with PEP-1, a HA binding inhibitor, exhibit a 30% reduction in intestinal epithelial stem cell proliferation and pronounced intestinal atrophy [38]. Longer duration of PEP-1 administration in adult mice was associated with shorter intestinal and colonic length, suggesting that intestinal homeostasis is driven, at least in part, by endogenous HA [35,38]. Intraperitoneal administration of high molecular weight (HMW) HA, from 3 to 8 weeks of age, led to hypoplasia of the small intestine and colon, further highlighting its potential role in intestinal development [38]. Here, we demonstrate that oral supplementation of the low molecular weight HA, HA 35 KDa, for 7 d in mouse pups promotes intestinal maturation and increases Paneth cell and goblet cell numbers compared to controls.

Rather than systemically administer HA, we used oral HA 35 KDa to determine its effects on the developing mouse intestine and microbiome. As a natural component of HM, with peak concentrations in term and preterm milk in the first two weeks of life, we hypothesized that HA affects intestinal development and maturation through the oral route. Secondly, oral HA likely reaches the small intestine intact given the lack of degradative enzymes in the mammalian digestive tract [58], which renders it a potential prebiotic capable of shaping the neonatal microbiome. The increase in goblet and Paneth cell numbers following HA 35 KDa supplementation is noteworthy. Paneth cells are pivotal for intestinal homeostasis through their role in maintaining the health of the stem cell niche and influencing the composition of the microbiome [59,60]. Goblet cells are particularly important in generating the mucus layer of the intestine, preventing interactions between pathogenic bacteria and the epithelium, and providing support for commensal bacteria. Importantly, both goblet cell and Paneth cell numbers are deficient in preterm infants, leading to a decrease in the ability of the intestinal epithelium not only to protect against pathogens but also to promote the development of a healthy microbiome [61].

The versatile roles of the intestinal microbiota include nutrient acquisition, energy regulation for the host, and intestinal barrier function, incorporating biological processes such as epithelial proliferation, mucin production, and antimicrobial compound production [62,63]. Dysbiosis of the gut microbiota has been linked to abnormal intestinal development, increased intestinal permeability, and intestinal inflammation, including NEC [64,65,66]. Evidence also suggests that prebiotic and/or probiotic use balances microbiome composition and can prevent intestinal inflammation and NEC [67]. Our data demonstrate 7 d HA 35 KDa supplementation in mouse pups was associated with an increased relative abundance of the firmicutes Lactobacillales Lactobacillus, Clostridiales Ruminococcaceae, Lachnospiraceae Coprococcus, as well as the Gram-negative Gammaproteobacteria Enterobacteriales. In contrast, these organisms were decreased in control animals, which had an increased abundance of Bacteriodales Rikenellaceae, Bacillales Staphylococcaceae, Actinomcetales Corynebacteriaceae, Clostridiales Eubacteriaceae, and Betaproteobacteria Burkoleriales. HA 35 KDa treatment also enriched for microbial functions related to glycosaminoglycan and purine nucleotide degradation, carbohydrate synthesis, and reduced predicted functions of cofactor synthesis and cell wall assembly. The observed microbiome changes are consistent with findings from Lee et al. who demonstrated that nanomolecular HA bound to bilirubin leads to a relative abundance of the protective bacteria *Clostridium* XIVα and *Lactobacillus* in a DSS-colitis model [68]. While beneficial microorganisms such as Lactobacillales Lactobacillus [69,70,71] and short-chain fatty acid-producing Clostridiales Ruminococcaceae and Lachnospiraceae Coprococcus [72,73,74] promote intestinal epithelial proliferation, maintain gut barrier function, and protect against a variety of intestinal pathologies, further investigation is warranted to explore the contributions of these bacteria to the intestinal morphological and functional effects observed in HA 35 KDa-treated pups. Additional experiments investigating intestinal development and maturation while utilizing antibiotics or germ-free mice in P14 pups would further our understanding of the role of the microbiome on the intestinal development and maturation observed in pups treated with HA 35 KDa.

To elucidate the effect of oral HA 35 KDa supplementation on the intestinal epithelium in normal conditions, we used an unbiased transcriptomic approach. Multiple DEGs playing crucial roles in intestinal epithelial homeostasis, proliferation, and differentiation were identified. For example, EGF and insulin receptor signaling pathways are known regulators of intestinal epithelial proliferation and barrier function, which may impact intestinal pathologies, including NEC [75,76,77,78,79]. The PI3K/AKT signaling pathway is also broadly involved in regulating intestinal epithelial cell growth and survival, proliferation, and apoptosis [80]. Another signaling pathway predicted to be activated with HA 35 KDa supplementation is mTOR, a highly conserved eukaryotic serine/threonine kinase. mTOR senses and responds to nutrients, energy, stress, and growth factors (such as IGF-1, insulin, and amino acids) through two downstream signaling complexes, mTORC1 and mTORC2 [39]. The physiological role of this particular signaling pathway was demonstrated in the small intestinal epithelium, under both homeostasis and during stress [81]. Sampson et al. showed that mTOR disruption using global or conditional knockout mice causes intestinal epithelial defects including ileal villus blunting, and decreased Paneth and goblet cells [82], likely due to impairment of mTOR-dependent translation or excessive protein turnover. In contrast, Zhou et al. [83] showed that mTORC1 activation is associated with increased intestinal epithelial proliferation and reduced goblet cell and Paneth cell differentiation in the mouse intestine. In our study, expression of Eif4e and 40S Ribo, direct downstream targets of mTORC1, was upregulated in HA 35 KDa-treated mouse pups compared to controls. HA 35 KDa-treated pups displayed increased staining intensity for the mTORC1 activation marker, phosphorylated ribosomal protein S6 in the intestinal epithelium, as compared to controls. Though it is reasonable to conclude that HA 35 KDa-induced mTOR activation is partly responsible for the intestinal epithelial changes observed in our study, further research is needed to confirm these findings using pharmacological and genetic approaches.

In summary, we found that supplementation with HA 35 KDa, a HM HA mimic, promotes intestinal proliferation, differentiation, and microbial compositional changes in early postnatal mouse life, with a particular impact on the differentiated secretory enterocytes, Paneth, and goblet cells. Further investigation into the role of HA 35 KDa for a longer duration and at different time points, as well as microbiome changes, and the epithelial- and immune-specific effects, are warranted.

## Data Availability

The data presented in this study are available on request from the corresponding author.

## Supplementary Materials

The following are available online at https://www.mdpi.com/article/10.3390/nu13062030/s1, Figure S1: Ingenuity pathway analysis results for predicted upstream regulators in the HA 35 KDa group compared to controls. Figure S2: Predicted upstream regulators activated or inhibited based on HA 35 KDa treatment as compared to control. Figure S3: Ingenuity pathway analysis results for DEG between HA 35 KDa and controls. mTor signaling, Table S1: Differentially expressed genes (DEG) in the 35kDa—treated pups compared to control. Table S2: Diseases and Functions in HA 35 KDa as Compared to control.
