# Supplementary figures and images for: Acceleration of Small Intestine Development and Remodeling of the Microbiome Following Hyaluronan 35 kDa Treatment in Neonatal Mice
Source: Nutrients. 2021 Jun 12;13(6):2030. doi: 10.3390/nu13062030 (PMC8231646; doi:10.3390/nu13062030)

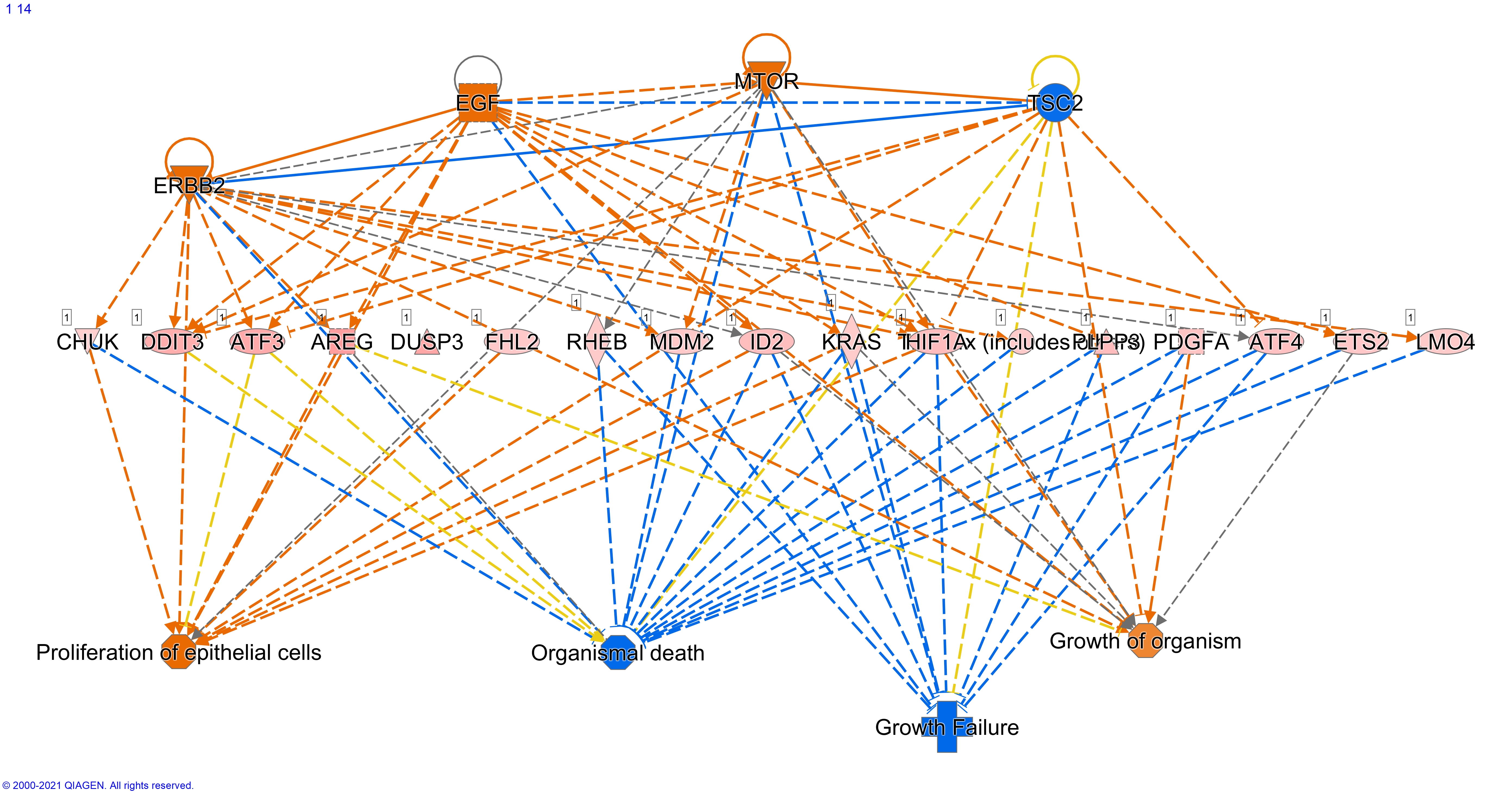

Supplement: Supplementary file 1 [file nutrients-13-02030-s001.zip › Supplementary Figure S1.jpg]

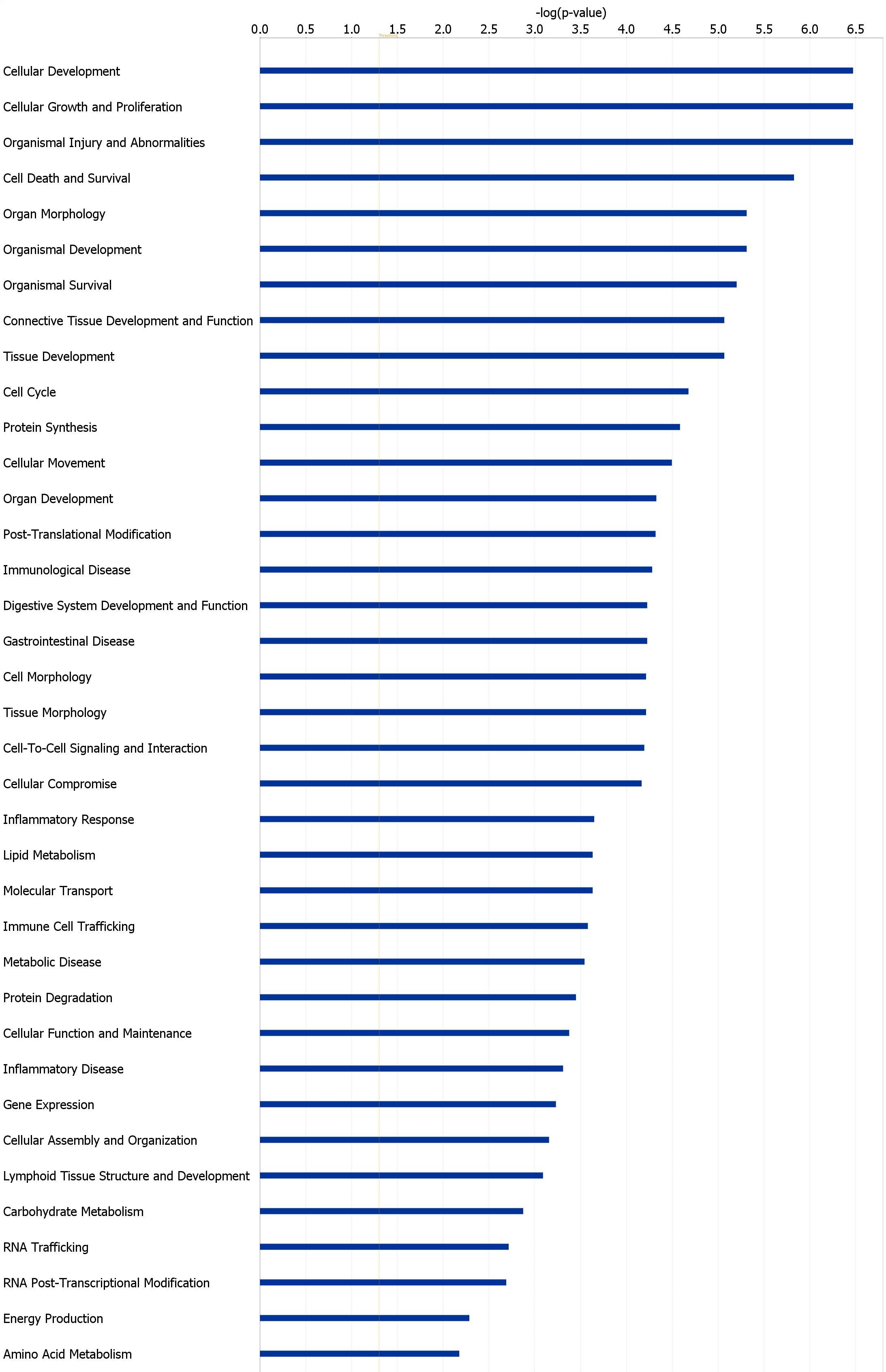

Supplement: Supplementary file 1 [file nutrients-13-02030-s001.zip › Supplementary Figure S2.tiff]

## Slide 1
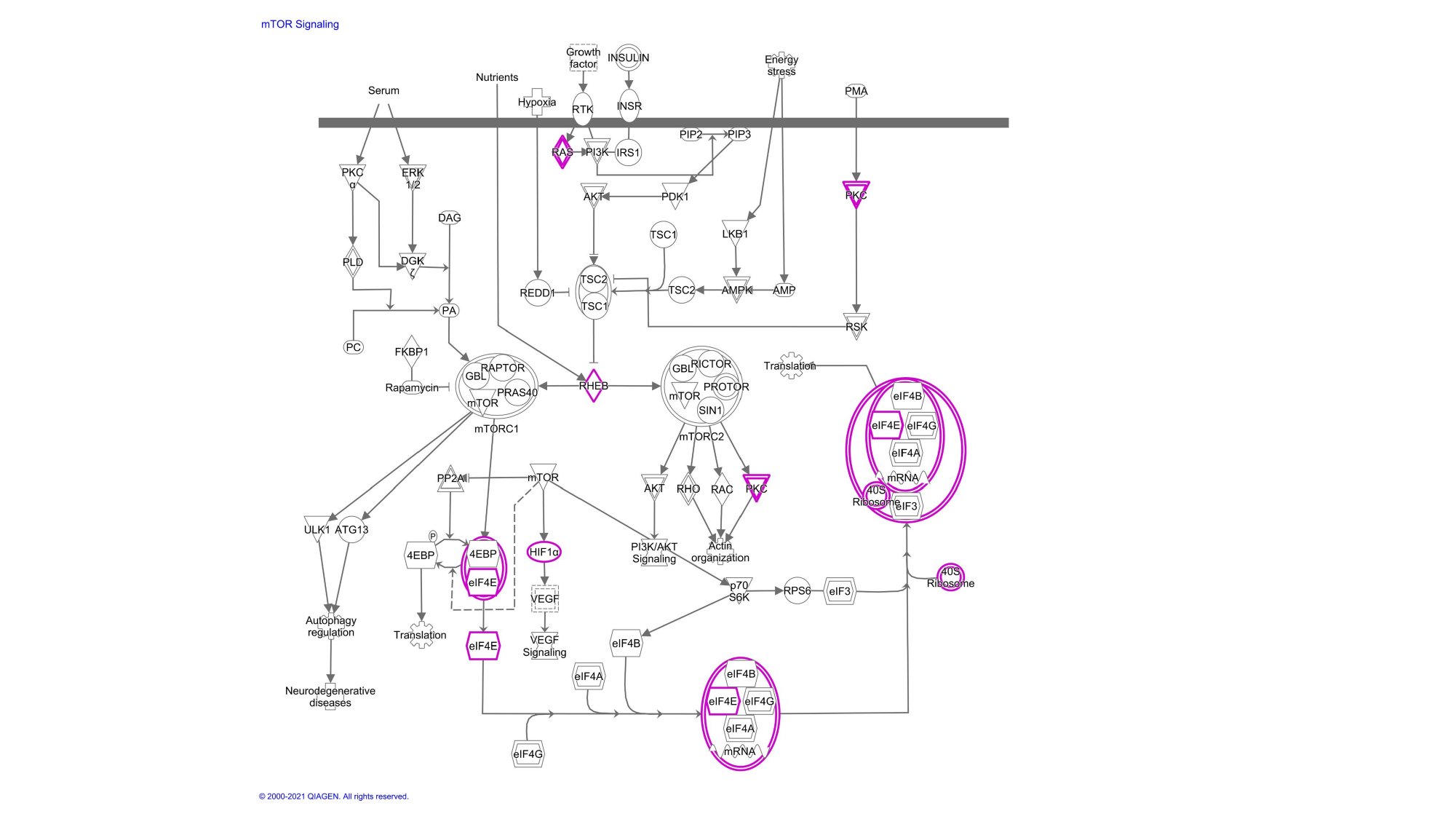

Supplement: Supplementary file 1 [file nutrients-13-02030-s001.zip › Supplementary Figure S3.pptx]
